# Risk factors and management of pasireotide-associated hyperglycemia in acromegaly

**DOI:** 10.1530/EC-20-0361

**Published:** 2020-10-29

**Authors:** Mônica R Gadelha, Feng Gu, Marcello D Bronstein, Thierry C Brue, Maria Fleseriu, Ilan Shimon, Aart J van der Lely, Shoba Ravichandran, Albert Kandra, Alberto M Pedroncelli, Annamaria A L Colao

**Affiliations:** 1 Endocrine Unit, Hospital Universitário Clementino Fraga Filho, Universidade Federal do Rio de Janeiro, Rio de Janeiro, Brazil; 2 Department of Endocrinology, Key Laboratory of Endocrinology, Ministry of Health, Peking Union Medical College Hospital, Beijing, China; 3 Neuroendocrine Unit, Division of Endocrinology and Metabolism, University of São Paulo Medical School, São Paulo, Brazil; 4 Aix-Marseille Université, Institut National de la Santé et de la Recherche Médicale, Marseille Medical Genetics, and Assistance Publique Hôpitaux de Marseille, Department of Endocrinology, Hôpital de la Conception, Centre de Référence des Maladies Rares de l’Hypophyse, Marseille, France; 5 Northwest Pituitary Center, Oregon Health & Science University, Portland, Oregon, USA; 6 Institute of Endocrinology and Metabolism, Rabin Medical Center, and Sackler School of Medicine, Tel-Aviv University, Petah-Tiqva, Israel; 7 Erasmus University Medical Center, Rotterdam, The Netherlands; 8 Novartis Pharmaceuticals Corporation, East Hanover, New Jersey, USA; 9 Recordati AG, Basel, Switzerland; 10 Dipartimento di Medicina Clinica e Chirurgia, Sezione di Endocrinologia, Università Federico II di Napoli, Naples, Italy

**Keywords:** pasireotide, acromegaly, hyperglycemia, diabetes mellitus

## Abstract

Pasireotide, a multireceptor-targeted somatostatin analog with highest affinity for somatostatin receptor subtype (SST) 5, has demonstrated superior efficacy over the SST_2_-preferential somatostatin analogs octreotide and lanreotide. The safety profile is similar to those of octreotide and lanreotide, except for a higher frequency and degree of hyperglycemia. This analysis investigated baseline characteristics and occurrence and management of hyperglycemia during pasireotide treatment in patients with acromegaly treated in two prospective clinical studies, SOM230C2305 (C2305) and SOM230C2402 (C2402; PAOLA). One hundred and seventy-eight patients naïve to medical therapy at baseline (C2305) and 125 uncontrolled on first-generation somatostatin analogs at baseline (C2402) received long-acting pasireotide in these studies. Of patients treated with pasireotide in studies C2305 and C2402, respectively, 75.3 (134/178) and 65.6% (82/125) developed hyperglycemia or experienced worsening of existing hyperglycemia. Occurrence of hyperglycemia during pasireotide treatment was less frequent in patients with lower age (<40 years, C2402; <30 years, C2305), normal glucose tolerance, and no history of hypertension or dyslipidemia at baseline. Thirteen (4%) patients discontinued pasireotide because of hyperglycemia-related adverse events. Metformin alone or in combination with other oral antidiabetic medications controlled elevations in glucose levels in most pasireotide-treated patients; 78% of C2305 patients and 73 (pasireotide 40 mg) and 60% (pasireotide 60 mg) of C2402 patients achieved the ADA/EASD goal of HbA_1c_ <7% (<53 mmol/mol) at the end of the core phase. Not all patients develop hyperglycemia, and it is reversible upon pasireotide withdrawal. Close monitoring, patient education and prompt action remain key elements in addressing hyperglycemia during pasireotide treatment.

## Introduction

Acromegaly is a chronic endocrine disorder typically caused by a benign tumor of the pituitary gland (1), which hypersecretes growth hormone (GH) with subsequent secretion of insulin-like growth factor I (IGF-I). Long-term excess of GH and IGF-I results in multiple comorbidities (2, 3), including hypertension and metabolic disturbances (4, 5), which contribute to increased mortality (6). Restoration of GH and IGF-I levels to normal levels is a key treatment goal in acromegaly and can reduce disease-associated morbidity and mortality (7, 8). Long-acting octreotide or lanreotide Autogel is the current standard of medical care for acromegaly (7). However, many patients do not achieve biochemical control with these agents (9).

Long-acting pasireotide, a multireceptor-targeted somatostatin analog (SSA), has been evaluated in the Phase III SOM230C2305 (C2305) (10) and SOM230C2402 (C2402; PAOLA) (11) studies. Patients enrolled in study C2305 had active acromegaly and had not received medical therapy at study entry (10), while patients in study C2402 were uncontrolled on first-generation SSAs prior to start of study (11). The primary endpoint for both studies was the proportion of patients with biochemical control (defined as GH <2.5 µg/L and normal IGF-I) at months 12 and 6, respectively. In study C2305, long-acting pasireotide treatment was associated with biochemical control in a significantly greater proportion of patients than long-acting octreotide (31.3% vs 19.2%; *P* = 0.007) at month 12; this difference was driven by greater reductions in IGF-I with pasireotide than with octreotide (10). The pronounced effect of pasireotide on IGF-I levels may be mediated not only by the inhibition of pituitary GH secretion but also through non-pituitary actions of pasireotide, including insulin suppression (12, 13). Similarly, in the C2402 study, significantly more patients on long-acting pasireotide 40 (15.4%; *P* = 0.0006) and 60 mg (20.0%; *P* < 0.0001) had biochemical control at month 6 than those who continued treatment with long-acting octreotide 30 mg or lanreotide Autogel 120 mg (0.0%). In both studies, the safety profile of long-acting pasireotide was generally similar to that of octreotide and lanreotide, except for the frequency and degree of hyperglycemia.

Pasireotide binds with high affinity to four of the five somatostatin receptor subtypes (SST): SST_1_, SST_2_, SST_3_ and SST_5_ (14), with highest affinity for SST_5_ (39-fold higher affinity than octreotide) (15). The efficacy of pasireotide in controlling GH and IGF-I levels is attributed to its activation of SST_2_ and SST_5_, which are most prevalent on somatotroph adenomas (16). Both of these receptors also play important roles in blood glucose regulation. Insulin secretion is mediated by both SST_2_ and SST_5_ (17), whereas glucagon secretion is mediated mainly by SST_2_ (18). As pasireotide has a higher affinity for SST_5_ than for SST_2_, insulin is suppressed to a greater degree than glucagon, which results in increased glucose levels (19). Indeed, pasireotide has been shown to reduce secretion of insulin, glucagon, glucagon-like peptide 1 (GLP-1) and glucose-dependent insulinotropic peptide in healthy volunteers (20). Thus, the reported effects of pasireotide on glucose homeostasis (10, 11) are likely the result of a decrease in both incretin response and insulin secretion (20).

Although the majority of patients receiving pasireotide will experience elevated glucose levels (hyperglycemia-related adverse events (AEs) were experienced by 57 and 64% of patients in C2305 (10) and C2402 (11), respectively), not all patients will develop hyperglycemia, and the magnitude of increase differs significantly between patients. The current work sought to investigate potential contributing factors to these differences in patients who participated in the C2305 and C2402 studies. Use of antidiabetic medications to manage hyperglycemia during pasireotide treatment was also assessed.

## Methods

### Study population and design

In study C2305, medical-therapy-naïve patients with active acromegaly (mean GH >5 µg/L or GH nadir ≥1 µg/L after an oral glucose tolerance test and IGF-I above the upper limit of normal (ULN)) were enrolled. Patients could have had previous pituitary surgery but no medical treatment, or were *de novo* with a visible pituitary adenoma on MRI (10). Patients were randomized in a double-blind manner to long-acting pasireotide 40 mg/28 days (*n* = 176) or long-acting octreotide 20 mg/28 days (*n* = 182). Uptitration to long-acting pasireotide 60 mg or long-acting octreotide 30 mg was permitted, but not mandatory, at months 3 and 7 if biochemical control (defined as GH <2.5 µg/L and normal IGF-I) was not achieved. The study consisted of two phases: a 12-month core phase and an optional extension phase in which patients achieving biochemical control or deriving benefit from treatment (investigator discretion) could continue their randomized treatment, otherwise they could cross over to the other treatment. Further study details have been published elsewhere (10).

In study C2402, patients with uncontrolled acromegaly (mean GH ≥2.5 μg/L and IGF­I >1.3 × ULN) following ≥6 months of long-acting octreotide 30 mg or lanreotide Autogel 120 mg monotherapy were randomized to long-acting pasireotide 40 mg (*n*=65), 60 mg (*n*=65), or continued treatment with long-acting octreotide 30 mg or lanreotide Autogel 120 mg (active control; *n*=68). Further study details have been published elsewhere (11).

Patients with inadequately controlled diabetes, as evidenced by glycated hemoglobin (HbA_1c_) >8% (>63.9 mmol/mol) despite treatment with antidiabetic medication(s), were not eligible to participate in studies C2305 and C2402. Patients with a known history or a new diagnosis of impaired fasting plasma glucose or diabetes mellitus were allowed to participate in each study; however, the study protocols stipulated that their blood glucose levels and antidiabetic treatment were to be closely monitored throughout these studies, with the latter adjusted as necessary.

Definitions of C2305 and C2402 subpopulations based on diabetic status are provided in the Supplementary material (see section on supplementary materials given at the end of this article). The number of patients deemed to have diabetes, pre-diabetes or normal glucose tolerance at baseline in this analysis of the C2402 study is different from the number classified as such in the primary publication of the study because of subtle differences in definitions between the two publications; in particular, the definitions of diabetes, pre-diabetes and normal glucose tolerance were updated for the current analysis to align them with guidelines set by the American Diabetes Association (ADA) for the classification of diabetes (Supplementary Table).

Studies C2305 and C2402 were conducted in accordance with the Declaration of Helsinki, and an independent ethics committee or institutional review board for each study site approved the relevant study protocol (see Supplementary material for full details). Consent was obtained from each patient or subject after full explanation of the purpose and nature of all procedures used.

### Hyperglycemia-related adverse events

Investigators were recommended to follow established guidelines by expert international diabetes organizations such as the ADA and the European Association for the Study of Diabetes (EASD). Hyperglycemia-related AEs were reported by the study investigators using various terms and included, but were not limited to, hyperglycemia (based on fasting glucose), diabetes mellitus, increased blood glucose, and type 2 diabetes mellitus. All AEs related to hyperglycemia were grouped together as hyperglycemia-related AEs. AEs were reported according to Common Terminology Criteria for Adverse Events (CTCAE) version 4.0.

### Definitions of antidiabetic medication categories

The efficacy of antidiabetic therapeutic regimens with regard to hyperglycemia during pasireotide treatment was assessed for both studies. Owing to the wide variety of antidiabetic medications that were used during the study periods, for the current analysis, three antidiabetic treatment groups were defined:

- ‘Metformin alone’ included patients who took at least one dose of metformin alone with no other oral antidiabetic (OAD) or insulin throughout the core phase
- ‘Metformin ± OAD’ included patients who received either metformin along with an OAD or any OAD other than metformin alone at any point during the core phase
- ‘Insulin ± OAD’ included patients who took insulin at any point during the core phase of the study with or without an OAD (which could have included metformin)

OADs included dipeptidyl peptidase 4 inhibitors, sulfonylureas, meglitinides, thiazolidinediones and alpha-glucosidase inhibitors. Only two patients, both in the C2402 study, received a GLP-1 receptor agonist (liraglutide); these two patients were not included in these analyses.

### Assessments and statistical analysis

All blood samples for assessment of fasting plasma glucose (FPG) and HbA_1c_ were taken after an overnight fast and before administration of long-acting pasireotide.

This study reports the baseline characteristics and occurrence and management of hyperglycemia in patients with acromegaly who were either naïve to medical therapy (C2305) or uncontrolled on first-generation SSAs (C2402) at baseline and were treated with pasireotide during the core phase of these studies. The occurrence and management of hyperglycemia in patients who crossed over from pasireotide to octreotide and vice versa in the extension phase of the C2305 study are also reported here. Descriptive statistics are presented for the current work. Further details on statistical analyses have been published elsewhere (10, 11). Univariate logistic regression analysis was performed on the safety analysis set (i.e. all patients who received at least one dose of study medication with a valid post-baseline safety assessment) to explore factors that might impact on hyperglycemia. *P* values provided are nominal. No multiplicity adjustments were performed; therefore, statistical interpretation should be made with caution.

## Results

### Baseline characteristics of patients who did not experience hyperglycemia during treatment with pasireotide

Overall, 75.3% (134/178) of pasireotide-treated patients in study C2305, and 65.6% (82/125) in study C2402, experienced hyperglycemia-related AEs during the core phases of both trials (Table 1). Younger patients, and those without diabetes or a history of hypertension or dyslipidemia at baseline, constituted most of the patients not experiencing hyperglycemia during the studies.

**Table 1:** Baseline characteristics and disease history of patients who did not and did experience hyperglycemia post-baseline while receiving pasireotide.

Hyperglycemia post-baseline	C2305		C2402			
	40 mg		40 mg		60 mg	
	No (*n* = 44)	Yes (*n* = 134)	No^a^ (*n* = 25)	Yes^a^ (*n* = 38)	No^a^ (*n* = 18)	Yes^a^ (*n* = 44)
Mean age, years (s.d.)	38.6 (11.5)	47.1 (11.9)	40.0 (14.1)	44.1 (14.0)	36.7 (9.4)	49.4 (14.5)
Age group, *n* (%)						
18–<30 years	12 (27.3)	8 (6.0)	6 (24.0)	7 (18.4)	6 (33.3)	4 (9.1)
30–<40 years	12 (27.3)	34 (25.4)	8 (32.0)	6 (15.8)	6 (33.3)	5 (11.4)
40–<50 years	10 (22.7)	30 (22.4)	5 (20.0)	12 (31.6)	4 (22.2)	17 (38.6)
50–<60 years	9 (20.5)	45 (33.6)	4 (16.0)	10 (26.3)	2 (11.1)	7 (15.9)
≥60 years	1 (2.3)	17 (12.7)	2 (8.0)	3 (7.9)	0	11 (25.0)
Mean BMI, kg/m^2^ (s.d.)	28.1 (4.9)	28.9 (4.5)	27.8 (4.2)	30.1 (5.4)	27.6 (4.7)	31.0 (6.6)
Baseline diabetic status, *n* (%)						
NGT	27 (61.4)	30 (22.4)	6 (24.0)	1 (2.6)	8 (44.4)	4 (9.1)
Pre-diabetic	10 (22.7)	58 (43.3)	9 (36.0)	2 (5.3)	6 (33.3)	7 (15.9)
Diabetic	7 (15.9)	46 (34.3)	10 (40.0)	35 (92.1)	4 (22.2)	33 (75.0)
Baseline FPG, *n* (%)						
<100 mg/dL (<5.6 mmol/L)	34 (77.3)	85 (63.4)	12 (48.0)	13 (34.2)	11 (61.1)	13 (29.5)
100–<126 mg/dL (5.6–<7.0 mmol/L)	8 (18.2)	43 (32.1)	11 (44.0)	20 (52.6)	6 (33.3)	22 (50.0)
≥126 mg/dL (≥7.0 mmol/L)	1 (2.3)	6 (4.5)	2 (8.0)	5 (13.2)	1 (5.6)	9 (20.5)
Missing data	1 (2.3)	0	0	0	0	0
Baseline HbA_1c_, *n* (%)						
<5.7% (<37.7 mmol/mol)	30 (68.2)	44 (32.8)	16 (64.0)	7 (18.4)	11 (61.1)	11 (25.0)
5.7–<6.5% (37.7–<47.5 mmol/mol)	12 (27.3)	75 (56.0)	8 (32.0)	21 (55.3)	7 (38.9)	19 (43.2)
6.5–<8.0% (47.5–<63.9 mmol/mol)	2 (4.5)	15 (11.2)	1 (4.0)	9 (23.7)	0	13 (29.5)
Missing data	0	0	0	1 (2.6)	0	1 (2.3)
Baseline mean GH, µg/L (s.d.)	16.2 (20.7)	23.5 (34.8)	25.6 (53.5)	12.5 (16.6)	10.3 (10.0)	13.4 (25.6)
Baseline mean IGF-I, × ULN (s.d.)	2.4 (1.1)	3.2 (1.3)	2.3 (0.8)	2.6 (1.1)	2.2 (0.8)	3.0 (1.2)
Mean time since diagnosis, months (s.d.)	15.1 (23.8)	21.7 (50.6)	49.9 (46.2)	76.9 (68.7)	61.2 (42.5)	80.1 (73.1)
History of hypertension						
No	36 (81.8)	73 (54.5)	21 (84.0)	26 (68.4)	16 (88.9)	21 (47.7)
Yes	8 (18.2)	61 (45.5)	4 (16.0)	12 (31.6)	2 (11.1)	23 (52.3)
History of dyslipidemia						
No	35 (79.5)	97 (72.4)	22 (88.0)	24 (63.2)	17 (94.4)	24 (54.5)
Yes	9 (20.5)	37 (27.6)	3 (12.0)	14 (36.8)	1 (5.6)	20 (45.5)

^a^This analysis of the C2402 study explored the effect of pasireotide on FPG and HbA_1c_ levels according to whether patients developed hyperglycemia at any time after initiation of pasireotide, whereas previous analyses of the study reported on this only for patients who had an evaluable assessment at the end of the core study (28).

FPG, fasting plasma glucose; GH, growth hormone; HbA_1c_, glycated hemoglobin; IGF-I, insulin-like growth factor I; NGT, normal glucose tolerance; ULN, upper limit of normal.

#### Age

Mean ages were similar across the two studies. Patients <30 years of age in study C2305, and <40 years of age in study C2402, were less likely to experience hyperglycemia with pasireotide than patients in older age categories (Table 1). However, within these age categories, the number of patients with diabetes at baseline varied: 7.5% (4/53) in study C2305 aged <30 years, and 37.8 (17/45) and 18.9% (7/37) in the pasireotide 40 and 60 mg treatment groups, respectively, in study C2402 aged <40 years. Of the diabetic patients at baseline in these younger age categories, 75.0% (3/4) in study C2305, and 70.6 (12/17) and 85.7% (6/7) in the pasireotide 40 and 60 mg treatment groups in study C2402, developed hyperglycemia while receiving pasireotide. Of the diabetic patients at baseline in the older age categories in studies C2305 (≥30 years) and C2402 (≥40 years), 87.8% (43/49) in study C2305, and 82.1 (23/28) and 90.0% (27/30) in the pasireotide 40 and 60 mg treatment arms in study C2402, developed hyperglycemia during pasireotide treatment.

#### Diabetic status

In studies C2305 and C2402, 68.0 (121/178) and 84.8% (106/125) of patients, respectively, were diabetic or pre-diabetic at baseline (Table 1). For patients with normal glucose tolerance (NGT) at baseline, 52.6% (30/57) of patients in study C2305, and 14.3 (1/7; 40 mg dose) and 33.3% (4/12; 60 mg dose) of patients in study C2402, experienced hyperglycemia while receiving pasireotide. The percentages of patients who were diabetic at baseline and who did not experience post-baseline hyperglycemia with pasireotide were 13.2% (7/53) for C2305 patients and 22.2 (10/45; 40 mg dose) and 10.8% (4/37; 60 mg dose) for C2402 patients.

#### Hypertension

In patients without a history of hypertension who received pasireotide, 67.0% (73/109) of C2305 patients, and 55.3 (26/47; 40 mg dose) and 56.8% (21/37; 60 mg dose) of C2402 patients, developed hyperglycemia (Table 1). Corresponding percentages for patients with a history of hypertension were 88.4 (61/69), 75.0 (12/16; 40 mg dose) and 92.0% (23/25; 60 mg dose) for patients in studies C2305 and C2402, respectively.

#### Dyslipidemia

In studies C2305 and C2402, respectively, 73.5 (97/132), 52.2 (24/46; 40 mg dose) and 58.5% (24/41; 60 mg dose) of patients without a history of dyslipidemia developed hyperglycemia with pasireotide treatment (Table 1). Corresponding percentages for patients with a history of dyslipidemia were 80.4 (37/46), 82.4 (14/17; 40 mg dose) and 95.2% (20/21; 60 mg dose).

### Risk factors for worsening glucose control

In both studies, diabetes mellitus and elevated HbA_1c_ at baseline were predictive risk factors for worsening of glucose control during treatment with pasireotide (Table 2). Additional risk factors identified were age, a history of hypertension in patients from study C2305, and a history of dyslipidemia and BMI ≥30 kg/m^2^ in patients from study C2402 (Table 2).

**Table 2:** Logistic regression analysis of possible risk factors for developing hyperglycemia.

Risk factor	C2305		C2402	
	Odds ratio (95% CI)	*P* value	Odds ratio (95% CI)	*P* value
Baseline age, years				
Per unit increase	1.04 (1.02, 1.05)	<0.0005	1.02 (1.00, 1.04)	0.113
≥40 vs 40	1.76 (1.12, 2.76)	0.015	2.25 (1.21, 4.20)	0.01
BMI, kg/m^2^				
25–<30 vs <25	1.13 (0.63, 2.04)	0.673	1.31 (0.58, 2.99)	0.516
≥30 vs <25	0.90 (0.49, 1.66)	0.735	2.65 (1.14, 6.16)	0.023
Years with disease				
Per unit increase	1.02 (0.96, 1.09)	0.534	1.02 (0.97, 1.08)	0.379
Baseline FPG, mg/dL (mmol/L)				
Per unit increase	1.01 (1.00, 1.03)	0.055	1.01 (1.00, 1.03)	0.149
100–<126 (5.6–<7.0) vs <100 (<5.6)	1.63 (0.99, 2.69)	0.055	0.97 (0.52, 1.82)	0.93
≥126 (≥7.0) vs <100 (<5.6)	1.58 (0.63, 3.97)	0.327	1.29 (0.55, 3.02)	0.561
Baseline HbA_1c_, % (mmol/mol)				
Per unit increase	2.79 (1.74, 4.50)	<0.0005	4.30 (2.14, 8.64)	<0.0005
5.7–<6.5 (37.7–<47.5) vs <5.7 (<37.7)	3.34 (2.03, 5.52)	<0.0005	2.37 (1.18, 4.77)	0.015
6.5–<8.0 (47.5–<63.9) vs <5.7 (<37.7)	3.23 (1.38, 7.53)	0.007	8.65 (2.76, 27.05)	<0.0005
6.5–<8.0 (47.5–<63.9) vs 5.7–<6.5 (37.7–<47.5)	0.96 (0.43, 2.14)	0.929	3.64 (1.34, 9.92)	0.012
History of diabetes mellitus				
Diabetic vs NGT	4.74 (2.66, 8.47)	<0.0005	4.80 (1.66, 13.91)	0.004
Pre-diabetic vs NGT	2.94 (1.73, 5.01)	<0.0005	1.21 (0.36, 4.11)	0.756
History of hypertension				
Yes vs no	2.38 (1.46, 3.88)	<0.0005	1.56 (0.87, 2.80)	0.133
History of dyslipidemia				
Yes vs no	1.83 (1.08, 3.12)	0.026	3.59 (1.83, 7.05)	<0.0005
Mean baseline GH, μg/L				
Per unit increase	1.01 (1.00, 1.02)	0.131	1.00 (0.99, 1.01)	0.772
Baseline IGF-I × ULN				
Per unit increase	1.00 (1.00, 1.00)	0.298	1.00 (1.00, 1.00)	0.361

FPG, fasting plasma glucose; GH, growth hormone; HbA_1c_, glycated hemoglobin; IGF-I, insulin-like growth factor I; NGT, normal glucose tolerance; ULN, upper limit of normal.

### Change in glycemic parameters by baseline diabetic status

**Figure 1:**
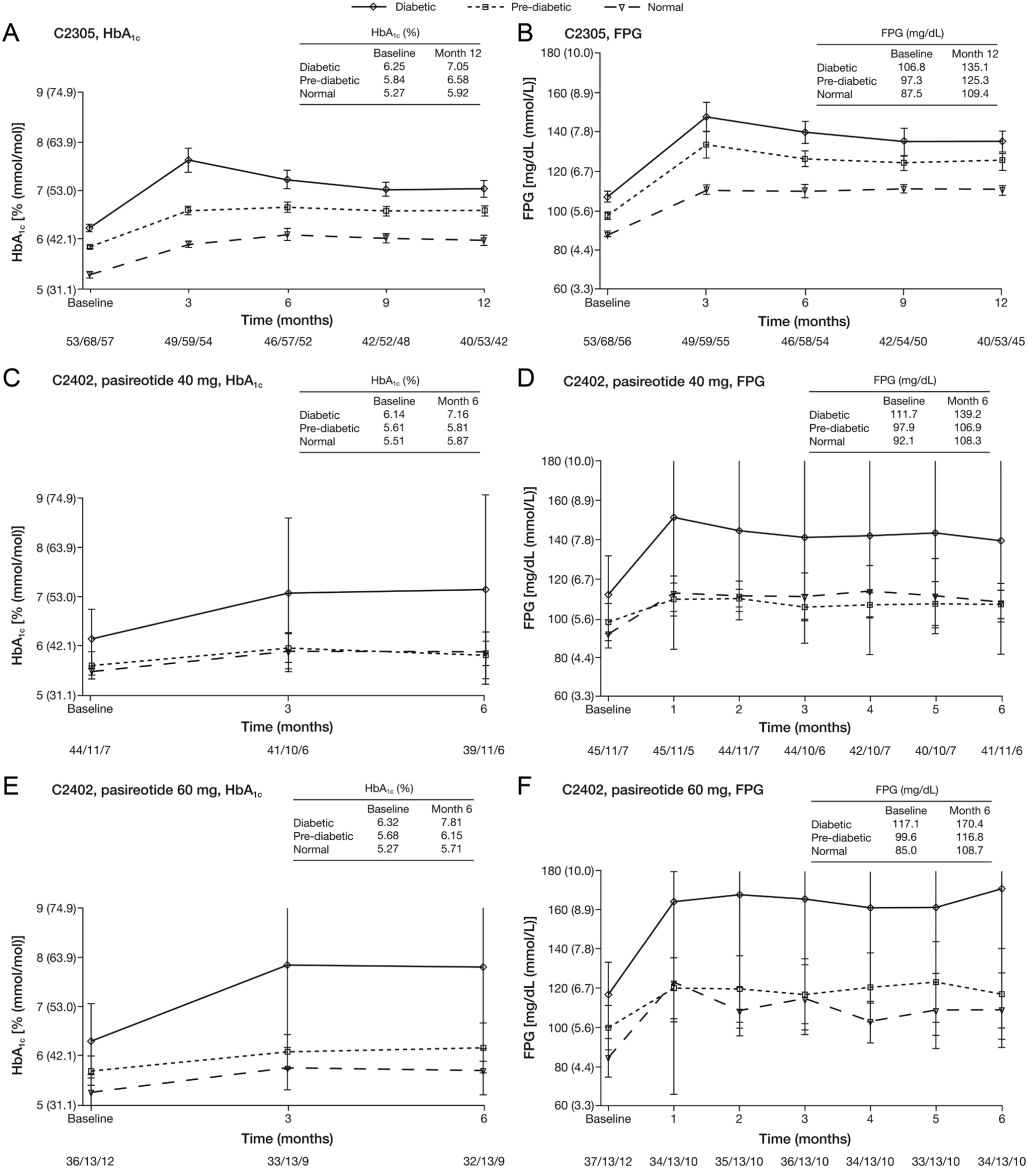
Mean HbA_1c_ and FPG over time by baseline diabetic status and pasireotide treatment in the core phases of studies C2305 and C2402. Data are presented as mean ± s.e. Numbers below the horizontal axes refer to numbers of patients in the diabetic/pre-diabetic/NGT groups. FPG, fasting plasma glucose; HbA_1c_, glycated hemoglobin; NGT, normal glucose tolerance.

In both study populations, those with diabetes at baseline receiving pasireotide had the greatest magnitude of increase in HbA_1c_ and FPG (Fig. 1) compared with pre-diabetic patients and those with NGT at baseline. Peak plasma glucose values following pasireotide therapy were observed at month 1 (C2402) and month 3 (C2305).

### Glycemic control

The HbA_1c_ values of individual patients were evaluated at month 12 (C2305) and month 6 (C2402), or at the last available time point for patients who discontinued treatment early. At the end of the core phases of both studies, 78.1% of C2305 patients and 73.0 (pasireotide 40 mg) and 59.7% (pasireotide 60 mg) of C2402 patients had HbA_1c_ values that met the ADA/EASD goal of <7% (<53.0 mmol/mol). In study C2305, 54.7% of patients who were diabetic at baseline had a last HbA_1c_ value of <7% (<53.0 mmol/mol) vs 84.9% of diabetic patients at baseline, while 62.2 (pasireotide 40 mg) and 37.8% (pasireotide 60 mg) of diabetic patients in study C2402 met the same goal (vs 84.4 and 73.0% of diabetic patients, respectively, at baseline).

The proportion of patients who achieved biochemical control of acromegaly (defined as GH <2.5 µg/L and normal IGF-I) was similar for patients who did and did not experience hyperglycemia on pasireotide: 31.6% vs 30.2% (C2305), 15.8% vs 14.8% (C2402, pasireotide 40 mg) and 18.2% vs 23.8% (C2402, pasireotide 60 mg) achieved biochemical control.

### Safety profile

In both study populations, few or no patients with pre-diabetes or NGT at baseline experienced grade 3 or 4 hyperglycemia-related AEs during the core phases of both studies (Table 3). Grade 3–4 hyperglycemia-related AEs were, however, more frequent in patients with diabetes at baseline.

**Table 3:** Incidence and severity of hyperglycemia-related AEs in patients receiving pasireotide, according to diabetic status.^a^

	Diabetic			Pre-diabetic			Normal glucose tolerance		
	C2305	C2402^a^		C2305	C2402^a^		C2305	C2402^a^	
	40 mg	40 mg	60 mg	40 mg	40 mg	60 mg	40 mg	40 mg	60 mg
*n*	53	45	37	68	11	13	57	7	12
Hyperglycemia-related AEs, *n* (%)	37 (69.8)	32 (71.1)	26 (70.3)	40 (58.8)	7 (63.6)	6 (46.2)	25 (43.9)	3 (42.9)	6 (50.0)
Grade 3–4 AEs, *n* (%)	10 (18.9)	7 (15.6)	9 (24.3)	4 (5.9)	0	0	2 (3.5)	0	0

^a^The number of patients deemed to be diabetic, pre-diabetic or have normal glucose tolerance at baseline in this analysis of the C2402 study is different from the number classified as such in the primary publication of the study because the definitions of diabetic, pre-diabetic and normal glucose tolerance are slightly different in this analysis compared with those in the primary publication; the definitions were updated for this analysis to align them with guidelines set by the American Diabetes Association for the classification of diabetes.

AE, adverse event.

Overall, only a small number of patients discontinued pasireotide because of hyperglycemia-related AEs (as judged by the investigator). In the core phases of studies C2305 and C2402 (pasireotide 40 and 60 mg), respectively, these AEs were: diabetes mellitus, *n* = 3 (1.7%), *n* = 1 (1.6%), *n* = 1 (1.6%) and hyperglycemia, *n* = 2 (1.1%), *n* = 1 (1.6%), *n* = 3 (4.8%). Additionally, in study C2305, one patient (0.6%) discontinued pasireotide because of increased HbA_1c_, and one patient (0.6%) because of type 2 diabetes mellitus.

### Management of hyperglycemia

**Figure 2:**
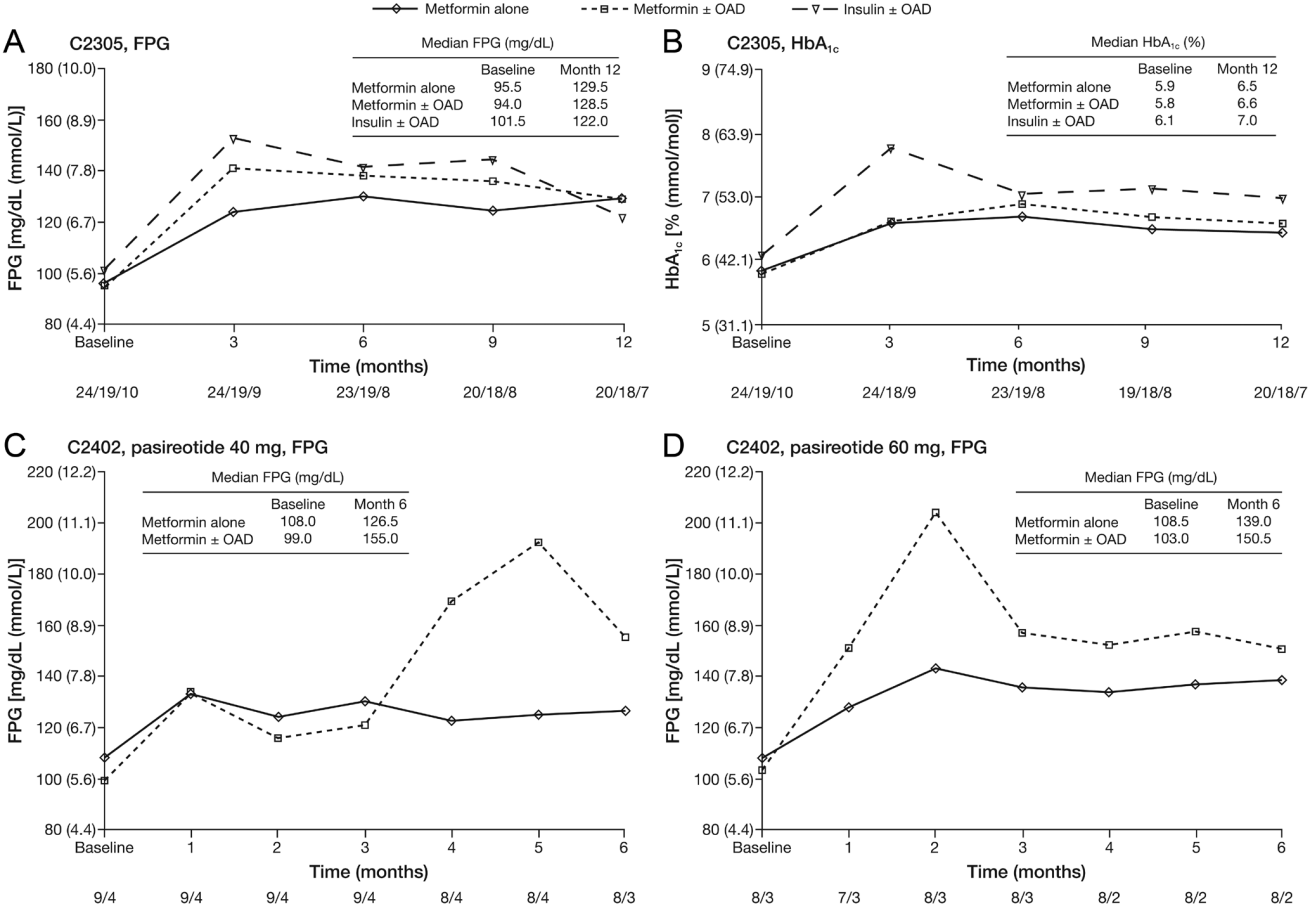
Median FPG and HbA_1c_ over time by antidiabetic treatment category for patients on pasireotide who did not have antidiabetic treatment at baseline and started antidiabetic intervention during the core phases of studies C2305 and C2402. Data presented as median values. Numbers below the horizontal axes refer to numbers of patients in the metformin alone/metformin ± OAD/insulin ± OAD groups in panels A and B, and the metformin alone/metformin ± OAD groups in panels C and D. In panels C and D, only two patients who had no antidiabetic medication at baseline in the pasireotide 60 mg group received insulin ± OAD during study C2402. This group is not shown because of difficulties interpreting the median for two patients. FPG, fasting plasma glucose; HbA_1c_, glycated hemoglobin; OAD, oral antidiabetic drug.

Patients in both trials who were not receiving antidiabetic therapy at study entry but who had antidiabetic medication initiated during the study were categorized into three analysis groups (metformin alone, metformin ± OAD, and insulin ± OAD) for exploratory assessment of FPG and HbA_1c_ levels at study baseline and over time by antidiabetic treatment group. In study C2305, baseline median FPG and HbA_1c_ values were similar in all three groups (Fig. 2A and B); following initiation of pasireotide, the greatest difference in median FPG and HbA_1c_ values between antidiabetic treatment groups occurred at month 3. In study C2402, median FPG levels were largely stabilized after 3 months with metformin-based antidiabetic medications (Fig. 2C and D). HbA_1c_ values increased from baseline to month 3 for both doses of pasireotide regardless of antidiabetic treatment group; however, only one patient had evaluable data at month 6.

### Reversibility of hyperglycemia

**Figure 3:**
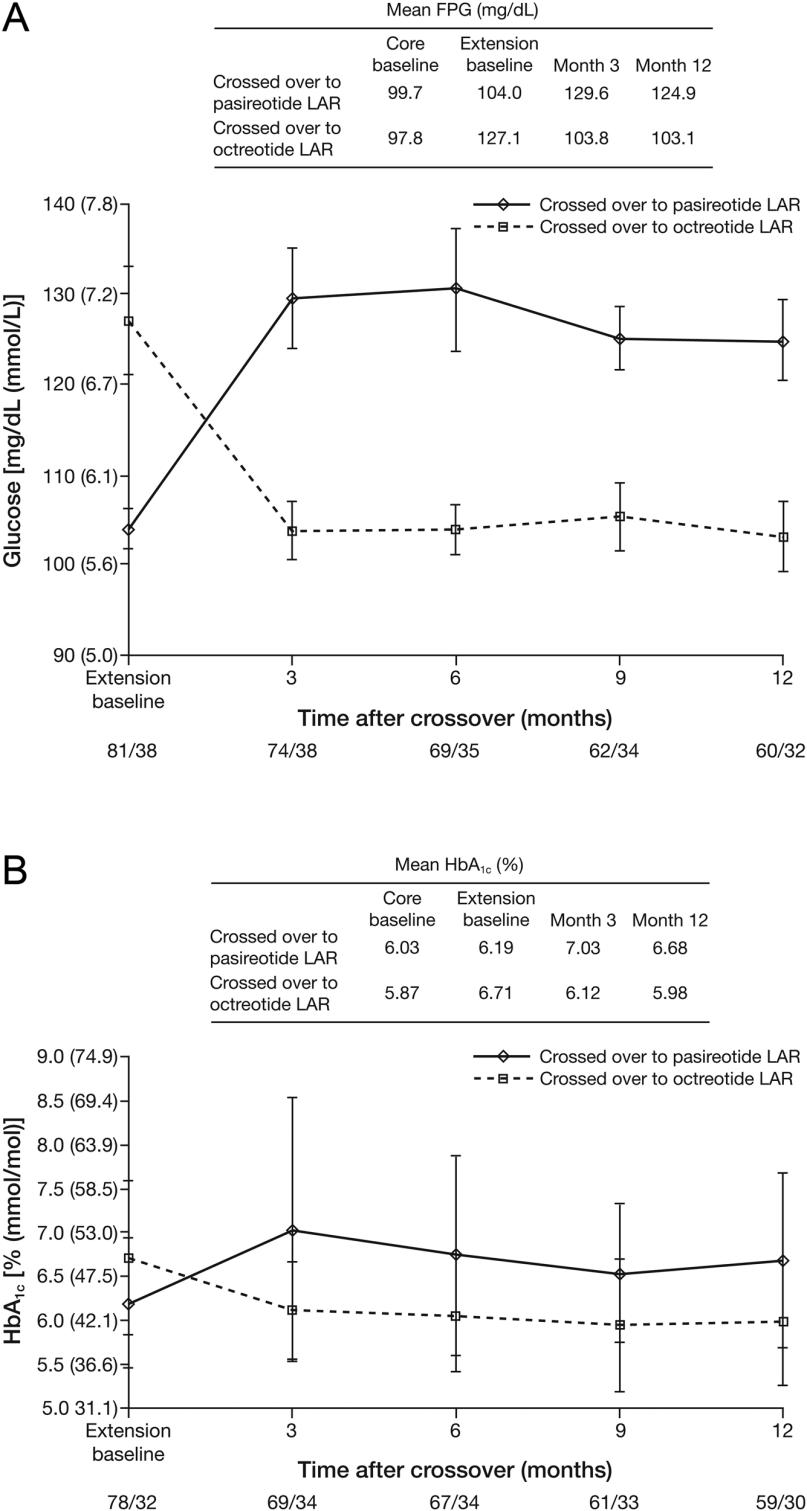
Change in (A) mean glucose levels and (B) mean HbA_1c_ after crossover in study C2305. Numbers below the horizontal axes refer to numbers of patients who crossed over to long-acting pasireotide/crossed over to long-acting octreotide. HbA_1c_, glycated hemoglobin; LAR, long-acting release.

The reversibility of elevations in glucose levels in patients receiving long-acting pasireotide is supported by a rapid decrease in glucose levels in patients who crossed over from long-acting pasireotide to long-acting octreotide in the extension phase of study C2305. Mean glucose levels were higher at extension baseline for patients who crossed over to octreotide (127.1 mg/dL (7.1 mmol/L)) than for those who crossed over to pasireotide (104.0 mg/dL (5.8 mmol/L)). By month 1 of the extension phase (the earliest time point measured), mean glucose levels in patients who crossed over to octreotide from pasireotide had decreased to 108.6 mg/dL (6.0 mmol/L) (Fig. 3). For patients who crossed over to octreotide, mean glucose and HbA_1c_ levels at 3 months post-crossover (103.8 mg/dL (5.8 mmol/L) and 6.1% (43.4 mmol/mol)) were similar to those observed at core baseline (97.8 mg/dL (5.4 mmol/L) and 5.9% (40.7 mmol/mol)), with subsequent stabilization to month 12 (Fig. 3).

## Discussion

Acromegaly is associated with a wide spectrum of morbidities (3, 4, 21, 22), with up to 75% of patients exhibiting disorders of glucose metabolism (5, 23, 24, 25). Consistent with these findings, 68 and 85% of patients enrolled in studies C2305 and C2402, respectively, who were treated with pasireotide had either diabetes or pre-diabetes at baseline. It was expected that the proportion of patients categorized as diabetic or pre-diabetic at study entry would be higher in patients enrolled in study C2402 because the time since diagnosis of acromegaly, and most likely the time of uncontrolled GH excess after diagnosis, at study entry was longer than in study C2305. Additionally, patients in study C2402 had been exposed to long-term treatment with first-generation somatostatin analogs, which may disturb glucose homeostasis (26, 27). These differences between the two patient populations could also contribute to different sensitivities in developing hyperglycemia while receiving pasireotide.

Pasireotide has demonstrated statistically significant and clinically relevant efficacy vs long-acting octreotide in medically naïve patients with active acromegaly, which is predominantly driven by greater reductions in IGF-I with pasireotide vs octreotide (C2305) (10), and vs long-acting octreotide/lanreotide Autogel in patients previously treated with these agents (C2402) prior to receiving pasireotide therapy (11). In both studies, suppression of GH and IGF-I levels was evident at month 3 and sustained thereafter (10, 11). The higher binding affinity of pasireotide for SST_5_ over SST_2_ vs octreotide or lanreotide not only accounts for its superior efficacy over these agents, but also the increased frequency and degree of hyperglycemia.

In a previous exploratory analysis of the C2402 study, occurrence of hyperglycemia while on pasireotide therapy was found to be more pronounced in patients with pre-existing glucose abnormalities (28). This finding is supported in the current analysis: patients with diabetes at baseline had the greatest magnitude of increase in HbA_1c_ following administration of pasireotide (vs patients with pre-diabetes or NGT). The maximal increase in mean FPG was observed at months 1 (C2402) and 3 (C2305) regardless of baseline diabetic status. In these two clinical trials, up to half of diabetic patients with acromegaly who received pasireotide were able to maintain glycemic control at target HbA_1c_ values recommended by international guidelines for the treatment of type 2 diabetes (29).

Patients with acromegaly who achieve biochemical control and whose comorbidities are appropriately managed benefit from the most favorable clinical outcomes (30), to the extent that mortality approaches that of the general population (31). In this context, the control of hyperglycemia during pasireotide treatment (occurring in ~75 and ~66% of patients in studies C2305 and C2402, respectively) is of paramount importance, given that type 2 diabetes is associated with increased risk of mortality (32). It is important to note, however, that pasireotide does not cause type 2 diabetes mellitus but secondary diabetes, which is reversible upon discontinuation of the drug causing the elevations in blood glucose. As clinical experience with pasireotide increases, management of instances of hyperglycemia is expected to improve even further. The present work demonstrates that metformin monotherapy or in combination with other oral antidiabetic medications, such as incretin-based dipeptidyl peptidase 4 inhibitors, effectively controlled elevations in glucose levels in most pasireotide-treated patients. Control of hyperglycemia as defined by the ADA/EASD (HbA_1c_ <7% (<53.0 mmol/mol)) at last available assessment was achieved in ~78% of patients in study C2305 and ~73 (pasireotide 40 mg) and ~60% (pasireotide 60 mg) of patients in study C2402. The UK prospective diabetes study demonstrated that any reduction in HbA_1c_ level in patients with diabetes is associated with a reduced risk of complications, with each 1% reduction corresponding to a 37% decreased risk of microvascular complications and a 21% decreased risk of any endpoint or death related to diabetes (33). Notably, results from two large trials (ACCORD and ADVANCE) showed no benefit with regard to cardiovascular outcomes by intensively treating patients with diabetes to obtain HbA_1c_ levels of <6.5% (<47.5 mmol/mol) (34, 35), informing the ADA/EASD recommendation of an HbA_1c_ level of <7% (<53.0 mmol/mol) for the control of diabetes (36). These results, combined with findings from our analysis, suggest that, with proactive monitoring and prompt management, pasireotide-associated hyperglycemia is unlikely to impact on long-term clinical outcomes, although further studies are warranted.

As only two patients were treated with a GLP-1 agonist in these two clinical trials, no information on the use of these agents in patients with acromegaly can be obtained. However, experience with managing pasireotide-related hyperglycemia in patients with Cushing’s disease has led to the following recommendations on managing elevated blood glucose levels in these patients. Hyperglycemia should be managed by initiation of metformin and staged treatment intensification with a dipeptidyl peptidase 4 inhibitor, with a switch to a GLP-1 receptor agonist and initiation of insulin, as required, to achieve and maintain glycemic control (37). Indeed, GLP-1 receptor agonists are often used in clinical practice to manage hyperglycemia during pasireotide treatment in patients with acromegaly.

Results from this study revealed that 66–75% of acromegaly patients treated with pasireotide developed hyperglycemia; 15–30% of patients achieved biochemical control on pasireotide, which was similar for patients who did and did not experience hyperglycemia. Although achieving biochemical control during pasireotide therapy did not appear to decrease the risk of hyperglycemia, younger age (<40 years), normal BMI, and the absence of comorbidities such as diabetes, hypertension, and dyslipidemia at baseline were associated with less likelihood of developing hyperglycemia. These findings do not imply, however, that older patients with comorbidities will necessarily experience hyperglycemia when treated with pasireotide, only that they are at higher risk.

Taking the aforementioned findings together with the low rate of discontinuations attributable to hyperglycemia-related AEs across both study populations, hyperglycemia during pasireotide treatment is manageable in most patients, even in those with pre-existing glucose abnormalities, as long as glucose levels are closely monitored. Notably, the C2305 and C2402 studies allowed for the inclusion of patients with a new diagnosis or known history of diabetes but not those with poorly controlled disease (HbA_1c_ >8% despite receipt of antidiabetic treatment). Importantly, increases in glucose levels during pasireotide treatment are reversible upon discontinuation of pasireotide therapy, supported by the decrease in glucose levels in patients who switched from long-acting pasireotide to long-acting octreotide in the extension phase of study C2305.

On a practical level, patients receiving pasireotide should be assessed for clinical benefit while on treatment. Blood glucose should be monitored in all patients, but with special attention given to patients with pre-existing impaired glucose tolerance or diabetes mellitus. If blood glucose levels increase, appropriate action should be taken, including the addition of antidiabetic medication. If hyperglycemia cannot be managed, discontinuation of pasireotide treatment can rapidly restore glucose levels to those seen prior to initiation of treatment.

The role of pasireotide in the acromegaly therapeutic continuum remains a key question for clinicians. Given the substantial burden of uncontrolled acromegaly and greater efficacy of pasireotide over first-generation SSAs (10, 11), pasireotide is an important treatment option for patients who remain uncontrolled on first-generation SSAs, the standard of medical care. Moreover, pasireotide may also be a useful treatment option in circumstances where alleviating tumor burden is strongly desired and surgery is not an option. The effect of pasireotide on reducing tumor volume in patients with acromegaly is well established (10, 38), and results of a recent subanalysis of a prospective open-label study (PAPE) provide additional insights into the antitumor effects of pasireotide. Of 47 patients treated with pasireotide in the study, hyperintensity of the tumor on T2-weighted MRI (typically indicative of cystic degeneration, tumor cell necrosis, or both) was observed in 10 patients within 9 months of treatment and in an additional four after the end of the study (median 29.5 months’ treatment) (39). Further studies evaluating whether the antitumor effects of pasireotide are sustained following pasireotide discontinuation are of great interest. Such studies may be especially important considering the potential for a few pasireotide recipients to develop hyperglycemia necessitating treatment cessation (~4% of patients in studies C2305 and C2402 combined).

In conclusion, increasing age and the presence of impaired glucose tolerance, dyslipidemia or hypertension appears to increase the likelihood of developing hyperglycemia during pasireotide treatment. However, hyperglycemia was manageable in most patients treated in studies C2305 and C2402, regardless of age or comorbidities present, with very few instances of these AEs necessitating discontinuation of pasireotide in the two studies.

## Supplementary Material

Risk factors and management of pasireotide-associated hyperglycemia in acromegaly

## Declaration of interest

M G has received speaker fees from Novartis, Ipsen and Pfizer, attended advisory boards for Novartis, and has been a principal investigator in clinical trials conducted by Novartis and Ipsen. T B has received institutional research support from Sandoz and Pfizer, and consultancy and lectureship fees from Novartis, Ipsen, Strongbridge and Pfizer. M F is the principal investigator on research grants to Oregon Health & Science University received from Chiasma, Ionis, Novartis and Pfizer, and an *ad hoc* scientific consultant to Chiasma, Ionis, Novartis and Pfizer. I S has received research grants, consulting and lectureship fees from Novartis, Chiasma and Pfizer. A C and A J v d L have received consultancy and speaker’s fees from Novartis, Pfizer and Ipsen. M D B has served on steering committees for Chiasma, Ipsen and Novartis, and has received speaker fees from Ipsen and Novartis, as well as clinical research grants from Ipsen and Novartis. F G has no conflicts of interest to disclose. S R, A K, and A M P are employees of Novartis.

## Funding

Both studies C2305 and C2402 were funded by Novahttp://dx.doi.org/10.13039/501100010450rtis Pharma AG.

## Data availability

Novartis is committed to sharing with qualified external researchers access to patient-level data and supporting clinical documents from eligible studies. These requests are reviewed and approved by an independent review panel on the basis of scientific merit. All data provided are anonymized to respect the privacy of patients who have participated in the trial, in line with applicable laws and regulations. This trial data availability is in accordance with the criteria and process described on www.clinicalstudydatarequest.com.
