# Supplementary material for: Risk factors and management of pasireotide-associated hyperglycemia in acromegaly
Source: Endocr Connect. 2020 Oct 29;9(12):1178–90. doi: 10.1530/EC-20-0361 (PMC7774766; doi:10.1530/EC-20-0361)
Supplement: Risk factors and management of pasireotide-associated hyperglycemia in acromegaly [file supplementary_material.pdf]

Risk factors and management of pasireotide-associated hyperglycemia in acromegaly

Supplementary material

Mônica R Gadelha, Feng Gu, Marcello D Bronstein, Thierry Brue, Maria Fleseriu, Ilan Shimon, Aart J van der Lely, Shoba Ravichandran, Albert Kandra, Alberto M Pedroncelli, Annamaria Colao

Definitions of C2305 and C2402* subpopulations based on diabetic status

Diabetic status at baseline	Definition	Definition of hyperglycemia
Diabetic[†]	Taking antidiabetic medication, or with prior history of diabetes mellitus, or with HbA$_{1c}$ ≥6.5% (≥47.5 mmol/mol) or FPG ≥126 mg/dL (≥7.0 mmol/L) or, for study C2402 only, 2-hour plasma glucose[‡] ≥200 mg/dL (≥11.1 mmol/L)	Percentage increase in FPG of >20% or percentage increase in HbA$_{1c}$ of ≥20% from baseline
Pre-diabetic	Not qualifying as diabetic and with FPG ≥100 and <126 mg/dL (≥5.6 and <7.0 mmol/L) or HbA$_{1c}$ ≥5.7% and <6.5% (≥38.8 and <47.5 mmol/mol) or, for study C2402 only, 2-hour plasma glucose[‡] 140–200 mg/dL (7.8–11.1 mmol/L)	FPG ≥126 mg/dL (≥7.0 mmol/L) or HbA$_{1c}$ ≥6.5% (≥47.5 mmol/mol) for the first time during the study
NGT	Not qualifying as diabetic or pre-diabetic and with FPG <100 mg/dL (<5.6 mmol/L) and/or HbA$_{1c}$ <5.7% (<38.8 mmol/mol) and, for study C2402 only, 2-hour plasma glucose <140 mg/dL (<7.8 mmol/L)	

*The criteria for defining diabetic status in this analysis of the C2402 study are the same as those used in the primary publication of the C2402 study (1) except for the inclusion of 2-hour plasma glucose values during an oral glucose tolerance test as an additional criterion in the current analysis,

in order to align the current analysis with guidelines set by the American Diabetes Association for the classification of diabetes; [†]Only diabetic patients used antidiabetic medication at baseline; [‡]During an oral glucose tolerance test at screening visit. FPG, fasting plasma glucose; HbA_{1c}, glycated hemoglobin; NGT, normal glucose tolerance

List of independent ethics committees and institutional review boards that provided ethics approval for the C2402 and C2305 studies

Study C2402

Ethik-Kommission der Ludwig-Maximilians-Universität (Munich, Germany), Friedrich-Alexander-Universität Erlangen-Nürnberg (Erlangen, Germany), Julius-Maximilians-Universität Würzburg (Würzburg, Germany), Commisie medsche ethiek (Leuven, Belgium), Comitê de Ética em Pesquisa em Seres Humanos da Faculdade de Ciencias Medicas- UNICAMP – SP (Campinas, Brazil), Comitê de Ética em Pesquisa da Faculdade de Medicina – USP (São Paulo, Brazil), Comitê de Ética em Pesquisa do Hospital Universitário Walter Cantidio (Fortaleza, Brazil), Comitê de Ética em Pesquisa da Unifesp/EPM (São Paulo, Brazil), Comitê de Ética em Pesquisa do Hospital Municipal Sao Jose (Joinville, Brazil), Comitê de Ética em Pesquisa da Faculdade de Medicina de Botucatu (Botucatu, Brazil), Comite de Etica en Investigacion con seres humanos – Fundacion Universitaria de Ciencias de la Salud (Bogota, Colombia), Comite de Etica en Investigación Centro Medico Imbanaco (Cali, Colombia), Helsinki Committee (Petah Tikva, Israel), Comitato Etico (Genoa, Italy), Comitato Etico (Naples, Italy), Comitato Etico Interaziendale (Turin, Italy), Comitato Etico Dell' Universita Cattolica del Sacro Cuore (Rome, Italy), Comitato Etico Scientifico (Messina, Italy), REK sør-øst (Oslo, Norway), Komisja Bioetyczna przy Akademii Medycznej we Wroclawiu (Wroclav, Poland), Komisja Bioetyczna przy Akademii Medycznej we Wroclawiu (Wroclav, Poland), Komisja Bioetyczna przy Akademii Medycznej we Wroclawiu (Wroclav, Poland), Comite Etica Investigacion Clinica (Alicante, Spain), Ceic Hospital Universidad Vall' De Hebron (Barcelona, Spain), CEIC Hospital Virgen del Rocio (Seville, Spain), Directorate of Ethics Advisory Committee (Ankara, Turkey), CPP Sud Est Mediterrannee II Marseille, (Marseille, France), NHS Southampton, (Bristol, England), Local Ethic Committee of Endocrinologic Scientific Center (Moscow, Russia), Ethic Committee under Association of Medical and Pharmaceutical Universities (Moscow, Russia), Local Ethic Committee of Altay Regional Clinical Hospital (Barnaul, Russia), Local Ethic Committee of Tyumen State Medical Academy (Tyumen, Russia), National Ethics Committee for Clinical Trial on Medicine (Bucharest, Romania), King Faisal Specialist

Hospital and Research Centre – Riyadh (Riyadh, Saudi Arabia), King Khlaid National Guard Hospital Jeddah (Jeddah, Saudi Arabia), Comite de Etica en Investigacion de Instituto Medica Especia Lizardo (Buenos Aires, Argentina), Western IRB (Olympia, WA, USA), Oregon Health & Science University (Portland, OR, USA), Medical School IRB (Ann Arbor, MI, USA), Southwestern Medical Center (Dallas, TX, USA), Comité d'éthique de la recherche sur I'humain – CHUS (Sherbrooke, Canada)

Study C2305

Comité de Ética en Investigación con Seres Humanos HSJ-FUCS (CEISH; Bogota, Colombia), Local Ethics Committee of the Federal State Institution "Endocrinology Research Center" of Rosmedtekhnologiy (Moscow, Russia), Inter-College Ethics Committee under the Association of Medical and Pharmaceutical colleges (Moscow, Russia), Ethic Committee of Medical Military Academy named after S.M. Kirov (Saint-Petersburg, Russia), Samsung Medical Center Institutional Review Board (Seoul, South Korea), Severance Hospital IRB (Seoul, South Korea), KyungHee University Medical center IRB (Seoul, South Korea), CEIC Hospital General Universitario de Alicante (Alicante, Spain), Comité Ético de Investigación Clínica de Galicia (La Coruña, Spain), Comité de Docencia e Investigación del Centro de Estudios Metabólicos y Endócrinos (Buenos Aires, Argentina), Comité de Docencia e Investigación Hospital Santa Lucía (Buenos Aires, Argentina), Comité de Revisión Institucional del Instituto Médico Especializado (IME; Buenos Aires, Argentina), General Hospital of Athens "G. Gennimatas", Scientific Council (Athens, Greece), Western Institutional Review Board (Puyallup, WA, USA), UCLA Office of Protection of Research Subjects (Los Angeles, CA, USA), Research Integrity office (Portland, OR, USA), The University of Texas M.D. Anderson Cancer Center Surveillance Committee (Houston, TX, USA), University of Michigan Institutional Review Board (IRBMED; Ann Arbor, MI, USA), Western Institutional Review Board (Olympia, WA, USA), Johns Hopkins Medicine Institutional Review Board Reed Hall (Baltimore, MD, USA), CPP Sud-Est IV – LYON Centre Régional de Lutte contre le cancer Léon Bérard (Lyon, France), Comités d'évaluation scientifique et d'éthique de la recherche (Montreal, Canada), Comité d'éthique de la recerche en santö chez l'humain (Fleurimont, Canada), Taichung Venterans General Hospital-The Institutional Review Board (Taichung, Taiwan), Comitato Etico dell'Azienda Ospedaliera Universitaria S.Martino di Genova (Genova, Italy), Comitato Etico Dell'universita' Cattolica Del Sacro Cuore, Policlinico Gemelli (Rome, Italy), Helsinki Committee (Petah-Tikva, Israel), IEC OF Peking Union Medical College Hospital (Beijing,

China), Istanbul Universitesi Capa Tip fakultesi Klinik Arastirmalar Etik Kurulu (Istanbul, Turkey), Atatürk Üniversitesi Tıp Fakültesi, İlaç Araştırmaları yerel Etik kurulu (Istanbul, Turkey), Comitê de Ética em Pesquisa da Faculdade de Medicina da Universidade de São Paulo (Sao Paulo, Brazil), Comitê de Ética em Pesquisa em Seres Humanos do Hospital de Clínicas da Universidade Federal do Paraná – HCUFPR (Curitiba, Brazil), Comitê de Ética em Pesquisa em Seres Humanos do Hospital Universitário Clementino Fraga Filho (Rio de Janeiro, Brazil), Comitê de Ética em Pesquisa em Seres Humanos do Hospital (Fortaleza, Brazil), Comitê de Ética em Pesquisa da Faculdade de Medicina da Universidade de Brasília - FM/UNB (Brasilia, Brazil)

Reference

1. Gadelha MR, Bronstein MD, Brue T, Coculescu M, Fleseriu M, Guitelman M, Pronin V, Raverot G, Shimon I, Lievre KK, Fleck J, Aout M, Pedroncelli AM & Colao A. Pasireotide versus continued treatment with octreotide or lanreotide in patients with inadequately controlled acromegaly (PAOLA): a randomised, phase 3 trial. *Lancet Diabetes Endocrinol* 2014 **2** 875-884.
